# SARS-CoV-2 Neutralization Resistance Mutations in Patient with HIV/AIDS, California, USA

**DOI:** 10.3201/eid2710.211461

**Published:** 2021-10

**Authors:** Seth A. Hoffman, Cristina Costales, Malaya K. Sahoo, Srikanth Palanisamy, Fumiko Yamamoto, ChunHong Huang, Michelle Verghese, Daniel A. Solis, Mamdouh Sibai, Aruna Subramanian, Lucy S. Tompkins, Philip Grant, Robert W. Shafer, Benjamin A. Pinsky

**Affiliations:** Stanford University School of Medicine, Stanford, California, USA

**Keywords:** SARS-CoV-2, HIV/AIDS, viral evolution, immunocompromised, COVID-19, respiratory infections, severe acute respiratory syndrome coronavirus 2, 2019 novel coronavirus disease, coronavirus disease, zoonoses, viruses, coronaviruses, California, United States, antimicrobial resistance

## Abstract

We report persistent severe acute respiratory syndrome coronavirus 2 infection in a patient with HIV/AIDS; the virus developed spike N terminal domain and receptor binding domain neutralization resistance mutations. Our findings suggest that immunocompromised patients can harbor emerging variants of severe acute respiratory syndrome coronavirus 2.

In December 2020, a 61-year-old woman living with HIV/AIDS was tested for severe acute respiratory syndrome coronavirus 2 (SARS-CoV-2) infection at a community testing center in California, USA; she produced an anterior nasal swab sample that tested positive by reverse transcription PCR (RT-PCR). At the time of sample collection, she had a 10-day history of nonproductive cough, and was not receiving antiretroviral therapy (Figure). Her CD4 count was 13 cells/μL and HIV-1 viral load was 262,000 copies/mL. She never required hospitalization for SARS-CoV-2 infection. Thirty days after symptom onset, she no longer had respiratory symptoms and underwent SARS-CoV-2 screening upon admission to Stanford Hospital (Stanford, CA, USA) for treatment of a severe decubitus ulcer. Her nasopharyngeal swab (NPS) sample tested positive for SARS-CoV-2 by RT-PCR (cycle threshold [C_t_] value 17.6). We detected minus-strand viral RNA, indicating active viral replication (*1*). On day 33, three days after admission to Stanford Hospital, antibody testing showed plasma positive for IgM against SARS-CoV-2 spike receptor binding domain (RBD) but negative for IgG against SARS-CoV-2 spike S1 (*2*,*3*). She began antiretroviral therapy (ART) 38 days after symptom onset. On day 45, an NPS sample tested positive (C_t_ 16.6) for SARS-CoV-2 with detectable minus-strand RNA.

**Figure:**
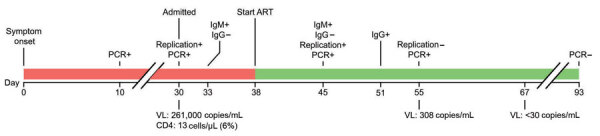
Timeline of SARS-CoV-2 infection in a patient with HIV/AIDS, California, USA. Line breaks indicate separation in time intervals. Replication indicates detection of minus-strand RNA. ART, antiretroviral therapy; IgG, spike S1 domain IgG; IgM, spike receptor binding domain IgM; VL, HIV viral load; +, positive; –, negative.

Because the patient had ongoing viral replication, we conducted whole-genome sequencing using archived nucleic acids from the NPS samples collected on days 30 and 45. We enriched the viral genome using laboratory-developed multiplex RT-PCR reactions that generated multiple overlapping amplicons of ≈1,200 bp. We prepared fragment libraries using NEBNext DNA Library Prep reagents (New England Biolabs, https://www.neb.com) according to the manufacturer’s instructions; we sequenced the libraries on Illumina MiSeq with single-end 150-cycle sequencing using MiSeq Reagent Kit v3 (https://www.illumina.com). We assembled the consensus sequences and identified mutations using a custom bioinformatics pipeline and SARS-CoV-2 isolate Wuhan-Hu-1 (GenBank accession no. NC_45512.2) as reference. For these 2 samples we observed mean whole-genome coverage of 963× (day 30) and 894× (day 45). We used the consensus sequences from the day 30 (GISAID accession no. EPI_ISL_2009056) and day 45 (GISAID accession no. EPI_ISL_2009057) samples to query the GISAID CoVserver (https://www.gisaid.org) and Phylogenetic Assignment of Named Global Outbreak LINeages (PANGOLIN, https://pangolin.cog-uk.io) to determine clade and lineage.

The sequence from the sample taken on day 30 revealed a G clade, B.1.234 lineage virus (Table). Because the day 45 sequence shares 18 mutations (8 synonymous and 10 nonsynonynmous) with the day 30 sequence and is the most closely related sequence to the day 30 sequence in GISAID, we believe the day 45 sequence probably evolved from the day 30 sequence. The day 45 sequence contained additional spike mutations, including C15F (variant percent 44.3%), del141_144 (17.5%), Y248N (13.4%), ins248_Y/LLSFN (44.5%), and E484Q (67.7%) (Table). The cysteine residue at position 15 (C15) in the spike N terminal domain (NTD) is linked by a disulfide bond to C136; mutations at either of these positions alter this bond and reduce neutralization by monoclonal antibodies (*4*). Deletions and insertions in the NTD are also involved in immune escape, including the common del141_144 mutation and insertions at position Y248 (*5*). The E484Q mutation is located in the RBD domain of the spike gene and is also found in the Kappa variant of interest (i.e., B.1.617.1) (*6*). Viruses harboring E484Q have reduced susceptibility to monoclonal antibodies, convalescent plasma, and vaccinee plasma (*7*,*8*).

**Table:** Mutations in severe acure respiratory syndrome coronavirus 2 sequences from a patient with HIV/AIDS, California, USA*

Nucleotide mutation	Translation	Variant reads/read depth (%)	
		Day 30	Day 45
Day 45 sample only			
21606G>T	S: C15F	ND	661/1,493 (44.3)
d21982_12	S: del_141–144	ND	248/1,420 (17.5)
22304T>A	S: Y248N	ND	333/2,488 (13.4)
ins_22304_12 (TAT>TTACTCAGTTTTAAT)	S: ins_248_Y->LLSFN	ND	919/2,065 (44.5)
23012G>C	S: E484Q	ND	1,088/1,607 (67.7)
Day 30 sample only			
26801C>T	Membrane protein: L93L	753/2,196 (34.3)	ND
27146A>G	Membrane protein: T208T	567/1,843 (30.8)	ND
Both samples			
241C>T	5′ untranslated region	1,206/1,213 (99.4)	848/851 (99.6)
829C>T	NSP2: N8N	862/866 (99.5)	652/653 (99.8)
2258G>A	NSP2: V485I	3,280/3,293 (99.6)	1,524/1,528 (99.7)
3037C>T	NSP3: F106F	1,781/1,783 (99.9)	1,139/1,142 (99.7)
6441A>G	NSP3: K1241R	2,385/2,389 (99.8)	1,499/1,500 (99.9)
8140C>T	NSP3: S1807S	2,491/2,505 (99.4)	1,399/1,410 (99.2)
9204A>G	NSP4: D217G	1,395/1,401 (99.6)	651/653 (99.7)
10015C>T	NSP4: Y487Y	527/530 (99.4)	244/245 (99.6)
10641C>T	NSP5: T196M	516/517 (99.8)	176/179 (98.3)
13858G>T	NSP12: D131Y (or RdRp D140Y)	3,814/3,832 (99.5)	3,081/3,100 (99.4)
14408C>T	NSP12: P314L (or RdRp P323L)	3,923/3,938 (99.6)	2,872/2,890 (99.4)
18288A>G	NSP14: V83V	2,498/2,521 (99.1)	1,868/1,886 (99.0)
20268A>G	NSP15: L216L	1,655/1,662 (99.6)	873/885 (98.6)
23403A>G	S: D614G	2,991/3,018 (99.1)	1,792/1,800 (99.6)
28744C>T	NP: I157I	5,669/5,704 (99.4)	4,311/4,343 (99.3)
28854C>T	NP: S194L	5,681/5,706 (99.6)	4,477/4,498 (99.5)
29384G>T	NP: D371Y	5,843/5,889 (99.2)	4,779/4,807 (99.4)
29445C>T	NP: T391I	5,928/6,006 (98.7)	4,641/4,670 (99.4)

*Compared with the reference Wuhan-Hu-1 (GenBank accession no. NC_045512.2) sequence. Patient samples collected 30 (hCoV-19/USA/CA-Stanford-07_S25/2021, GISAID accession no. EPI_ISL_2009057) and 45 (hCoV-19/USA/CA-Stanford-07_S24/2021, GISAID accession no. EPI_ISL_2009056) days after symptom onset. ND, not detected; NP, nucleoprotein; NSP, nonstructural protein; RdRp, RNA-dependent RNA polymerase; S, spike protein.

The patient showed SARS-CoV-2 IgG seroconversion on day 51, thirteen days after initiating ART. SARS-CoV-2 antibody isotypes typically follow a similar time-course; IgM, IgA, and IgG usually become detectable ≈14 days after illness onset (*2*). This patient’s delayed IgG class switch was probably caused by HIV/AIDS-associated B-cell dysfunction; we hypothesize that the ineffective IgM response might have selected for the observed spike mutations (*9*). On day 55, seventeen days after initiating ART, the patient’s HIV-1 viral load was 330 copies/mL. An NPS sample collected that day was negative for minus-strand SARS-CoV-2 RNA, and the viral load had decreased >1,000-fold (C_t_ 27.2). The patient’s SARS-CoV-2 infection remained asymptomatic throughout her hospitalization. On day 93, she produced an NPS sample that tested negative for SARS-CoV-2 RNA.

In summary, we describe an HIV-positive patient who had a prolonged course of asymptomatic, active SARS-CoV-2 infection leading to the emergence of NTD and RBD mutations associated with reduced antibody neutralization. Our findings add to the accumulating evidence that immunocompromised persons, including persons living with HIV/AIDS, might host ongoing SARS-CoV-2 replication that could enable the development of variants of concern/interest (F. Karim, unpub. data, https://www.medrxiv.org/content/10.1101/2021.06.03.21258228v1). The emergence of multiple spike mutations in this patient over a relatively short timeframe (i.e., 15 days) further highlights the potential role of persons living with uncontrolled HIV as possible sources of SARS-CoV-2 variants. Finally, these findings emphasize the need to diagnose HIV in the >6 million infected persons worldwide who are unaware of their status and provide them with accessible ART. These interventions are critical for overall global health and might also contribute to controlling the COVID-19 pandemic.
